# On the Behavioral Side of Procrastination: Exploring Behavioral Delay in Real-Life Settings

**DOI:** 10.3389/fpsyg.2018.00746

**Published:** 2018-05-16

**Authors:** Frode Svartdal, Sjur Granmo, Fredrik S. Færevaag

**Affiliations:** ^1^Department of Psychology, UiT The Arctic University of Norway, Tromsø, Norway; ^2^Faculty of Health and Welfare, Østfold University College, Fredrikstad, Norway; ^3^Bjørknes University College, Oslo, Norway

**Keywords:** procrastination, delay, dilatory behavior, behavioral measures, procrastination scale

## Abstract

This paper examines how procrastinators behave differently from non-procrastinators in implementing intended behavior. By focusing on time-related attributes of behavior, we demonstrate in five studies (aggregated *N* = 965) that onset delay seems to be a preferred option for procrastinators in common daily situations. Thus, when an action possibility is available for intended behavior, procrastinators tend to delay behavior onset, both in actual behavior and in onset preferences, often instigating chains of events with negative consequences. We discuss possible mechanisms responsible for such delays and explore how such mechanisms generate and sustain dilatory behavior. We conclude that a better understanding of why behavioral delays occur in early phases of action implementation is of importance in understanding and preventing procrastination.

## Introduction

Procrastination involves unnecessary and unwanted delay, be it decisional, implemental, or lack of timeliness (Lay, 1986; McCown et al., 1989; Mann et al., 1997; Steel, 2010). Furthermore, Steel (2007) emphasized that a core characteristic of procrastination is the realization by the actor that one will be worse off because of the delay. Hence, procrastination can be seen as irrational behavior—delaying some intended course of action, realizing that it is disadvantageous (Klingsieck, 2013). Behavioral delay in procrastination is observed in at least two ways. First, during action implementation, the person may divert to an alternative and more tempting course of action (Tice et al., 2001), indirectly delaying the original plan. Second, in a longer time perspective, the negative consequences of such diversions become visible, as for example when people postpone seeing their doctors until treatment is no longer an option (Worthley et al., 2006), or postpone the initiation of personal retirement plans (Byrne et al., 2006). In a longitudinal study, Tice and Baumeister (1997) demonstrated both forms of delay in a student sample. Students procrastinating early in the semester created a stress-free and pleasant situation for themselves, only to experience that these short-time benefits had long-term costs at the end of the semester.

Although the core problem of procrastination is behavioral delay, studies such as those discussed are in the minority in the procrastination literature. Most studies of procrastination have instead focused on self-reported delay as measured by procrastination scales and inventories (Steel, 2007; Rozental and Carlbring, 2014; Svartdal and Steel, 2017). An obvious motivation for this preference is that dilatory behavior is inherently subjective, making it reasonable to classify a given course of action as dilatory or not depending on the person’s intention, which is conveniently assessedby self-report. Another reason for favoring measurement scales in procrastination research is that dilatory behavior is often difficult to operationalize, as it is characterized by *not* occurring (given a plan). Again, resorting to self-reported deviations from plans is a convenient solution (e.g., Krause and Freund, 2014).

However, reliance on self-reported delay has moved procrastination research away from the core characteristic of procrastination, behavioral delay. As will be discussed, the number of studies focusing on behavior in procrastination research is scarce. Furthermore, reliance solely on self-reported procrastination may bias results. Notably, self-reported procrastination lacks a calibration mechanism that may help differentiate between trivial but harshly judged procrastination and more serious forms (e.g., Gröpel and Steel, 2008; Svartdal and Steel, 2017), which again has implications for prevalence estimates (e.g., Rozental and Carlbring, 2014). Third, as existing procrastination scales often address domain- and culture-specific behavior themes (e.g., Christmas shopping; cf. Lay, 1986), conclusions may be vulnerable to personal, cultural, and contextual variability (Svartdal et al., 2016). Hence, bringing back behavior into the procrastination equation may be worthwhile for a number of reasons.

In the present paper, we attempt to do so by focusing on behavioral delay when action possibility presents itself. Thus, rather than addressing the common measure of behavioral delay, lateness/timeliness in completing intended behavior (McCown et al., 1989; Tice and Baumeister, 1997), we address the implementation phase of intended action when the person can choose swift vs. delayed action. Such a focus on promptness (e.g., Schouwenburg, 1995) allows for a focus on time-related behavioral dimensions with less emphasis on *what* people are procrastinating, but stronger emphasis on *how* people behave when procrastinating. Looking forward, we argue that such a shift better captures important properties of behavioral onset delay seen in procrastination.

We start with a short discussion of behavioral delay and of possible models that explain the ontogeny of behavioral delay. Then we briefly examine existing literature on dilatory behavior in procrastination, demonstrating that there are surprisingly few studies examining the relation between self-reported procrastination and corresponding behavioral delay, and in particular onset delay. Finally, we report five studies that illuminate how onset delay manifests itself in procrastination.

### Models for Understanding Behavioral Delay

Analyzing procrastination from an evolutionary life history perspective, Chen and Chang (2016) argued that the procrastinator lives by a fast life strategy with a psychological time orientation on the present. Such a fast life strategy has been functional in unpredictable environments during evolution, fostering impulsivity, high risk-taking, overlooking consequences, and discounting the future. However, as contemporary life emphasizes planning, personal control, and accountability, a fast life strategy has become maladaptive. Accordingly, research has amply documented that procrastination is closely linked to impulsivity (van Eerde, 2003; Steel, 2007; Gustavson et al., 2014), with a preference for instantly gratifying options rather than more beneficial longer-term goals. Such a preference is associated with negative consequences that make habitual procrastination maladaptive. Thus, procrastination is associated with a number of adverse states and problems, including increased stress, lower task performance, reduced well-being, regret and suffering, and risk of mental and physical illness (Tice and Baumeister, 1997; Steel, 2007; Klingsieck, 2013; Steel and Ferrari, 2013; Sirois, 2014).

As impulsivity suggests a preference for immediate outcomes (Sharma et al., 2014; Steel and Weinhardt, 2017), but procrastination is characterized by delay, identification of mechanisms that can mediate the relation between impulsivity and behavioral delay is crucial. A common answer to this dilemma is that impulsive diversions to more attractive alternatives occur during implementation of plans, indirectly creating delays in realizing them (e.g., Schouwenburg, 1995; Tice et al., 2001; Steel, 2007). This may be seen as temporal discounting in that salient and immediately available rewards dominate over distant rewards (e.g., Steel and Weinhardt, 2017). Immediate rewards may be situational, but not necessarily. For example, taking a break from working with aversive, stressful, or difficult tasks, gaining swift mood repair, stress relief, and satisfaction (e.g., Tice and Bratslavsky, 2000; Tice et al., 2001; Sirois, 2007) may be highly rewarding, resulting in a direct form of task delay in that the primary motivation is to stop ongoing behavior rather than switching to something more attractive in the situation.

Temporal discounting of negative stimuli is also a possible mechanism causing behavioral delay. Response cost may serve as an example. As the cost of immediate action is more salient than the perceived cost of future action, behavioral delay may occur because the cost of performing it now seems higher than performing it later (e.g., Akerlof, 1991). A related and probably more important mechanism is avoidance of aversive tasks, preventing the occurrence of negative feelings, stress, and other forms of aversive states, resulting in avoidant styles of functioning (e.g., Díaz-Morales et al., 2008) and subsequent delays in task execution.

Note that the mechanisms discussed here imply delayed task execution; for all but the first mechanism, task delay should be reflected in reduced behavioral vigor directly. The mechanisms discussed are summarized in **Table 1**. We are not aware of prior research examining the effects of these mechanisms on behavioral onset delay specifically, but predictions are quite straightforward. First, the overall effect of these mechanisms should be delay in the execution of planned behavior, increasing the possibility of passivity, hesitation, and lingering. Second, it is likely that repeated occurrences involving one or several of these mechanisms may have established learned habits and reactions that themselves can cause delay. In both cases, when facing action possibility, it is likely that the procrastinator will respond with hesitation and lack of promptness. This response should be readily observable, differentiating procrastinators from non-procrastinators in situations where prompt action is possible and often advantageous. For the procrastinator, this response has most probably also generalized, so that delay is the default response in a variety of situations and modalities. Thus, when facing an action possibility, a “delay” rule should not only reveal itself in behavior but also in behavioral onset preferences. Although some situations should be especially prone to triggering delay (e.g., aversive or boring situations) and some not (e.g., situations with positive valence) (Steel, 2007), it is likely that the “delay” rule will be a default and automatic response in ordinary, everyday situations, potentially instigating a chain of events with negative long-term consequences for the procrastinator.

**Table 1:** Common contexts for behavioral delay.

Context	Action
Experiencing aversive, difficult, boring task	Diversion to something more attractive in the situation (engaging in competing attractive activities)
Experiencing aversive, difficult, boring task	Escape from aversive, stressful situation (immediate reduction of aversiveness, stress)
Expecting aversive, difficult, boring task	Avoidance (not experiencing aversiveness, stress)

### Research on Implemental Delay and Its Relation to Self-Reported Procrastination

As the focus of this paper is on delayed onset of intended behavior, we examined the procrastination literature for research concerning this issue specifically. We also traced research that address the relation between dilatory behavior and self-reported procrastination more generally. As seen in **Table 2**, we have identified only seven studies that address the self-reported procrastination–behavior relation explicitly, and only three address onset delay specifically (Senécal et al., 1997; Steel et al., 2001; Moon and Illingworth, 2005). Senécal et al. (1997) presented participants with a series of tasks differing in dimensions related to motivation (e.g., boring/difficult vs. interesting/easy), and measured (a) time to start the boring/difficult task, and (b) time to complete all tasks. For participants (students) expecting an evaluation of performance, onset delay of the boring/difficult task was markedly higher for high vs. low procrastinators, as was the total time to complete all tasks. However, the difference in implemental delay seemed to be due to the fact that high procrastinators not expecting to be evaluated demonstrated much shorter delays, even much shorter compared to low procrastinators (Senécal et al., 1997, Figure 1), making an inference from this study somewhat difficult. Steel et al. (2001) examined the intention-action gap and found that procrastinators differed from non-procrastinators only at the beginning and at the end of the course, and in the predicted directions: Procrastinators did less work than intended in the start of the semester, and more toward course completion. Finally, Moon and Illingworth (2005) obtained repeated behavioral measures of student procrastination (time from opening for tests to be taken to actual test time in repeated test windows throughout the semester) and found moderate correlations between a dispositional procrastination score and test onset delay. However, the pattern of procrastination throughout the semester did not differ between high and low procrastinators. Thus, although the students might differ in their initial levels of procrastination, all followed the same procrastination pattern over the course of the semester (Moon and Illingworth, 2005, p. 306).

**Table 2:** Studies assessing the self-reported procrastination–behavior relation.

	Theme	Measures
Tice and Baumeister, 1997	*Timeliness*: Procrastination scores correlate with delay in assignment submission	B, SR
Lay, 1986	*Timeliness*: Procrastinators overrepresented in delay mail return of inventories	B, SR
Howell et al., 2006	*Timeliness*: Submission pattern conform to a hyperbolic function, most submissions <10 h before deadline	B, SR
Senécal et al., 1997	*Onset delay*: Onset delay of boring/difficult task longer in high procrastinators, but only for students expecting to be evaluated	B, SR
Steel et al., 2001	*Onset delay*: Intention-action gap in procrastinators high at course start, but low at semester end	B, SR
Moon and Illingworth, 2005	*Onset delay*: Moderate correlations between dispositional procrastination score and test onset delay	B, SR
Kroese et al., 2014, 2016	*Bedtime procrastination*: Getting to bed later than planned “while no external circumstances prevent a person from doing so”	B, SR
Stead et al., 2010	*Delay in help-seeking*: Higher in procrastinators	SR
Sirois, 2007	*Risk willingness*: Correlates 0.45 with procrastination score	SR
Roig and DeTommaso, 1995; Clariana et al., 2012; Patrzek et al., 2015	*Academic misconduct*: Procrastination scores predict academic misconduct, e.g., cheating	SR
van Hooft et al., 2005	*Job search in unemployed*: Procrastination score related to self-reported job search behavior	SR
Tice et al., 2001	*Temptation*: Participants in a bad mood with fun distractors available, procrastinated the most	B
McCrea et al., 2008	*Mindset*: Concrete representation of a task leads to earlier execution of it	B

*B, procrastinating behavior; SR, self-reported procrastination/behavior.*

**Table 2** also includes studies that have assessed the self-reported procrastination–behavior relation by including a measure of self-reported behavior rather than observed behavior. Further, some studies that experimentally demonstrate procrastination have often done so without connecting behavioral procrastination to dispositional procrastination as measured by scales. Two examples are listed in the table. Note also that some studies not included in the table address behavioral measures of procrastination indirectly by comparing self-reported planned behavior to self-reported actual behavior, thereby obtaining a difference score used as a proxy for behavioral procrastination (e.g., Krause and Freund, 2014). Other studies have linked dispositional procrastination score to a product of behavior (e.g., grades; Steel et al., 2018).

The studies listed in **Table 2** assume a relatively close relation between dispositional procrastination and dilatory behavior documented by self-report or by actual behavioral delay (e.g., lateness in submitting reports). Overall, this expectation has gained support, but some qualifications should be noted. First, as noted, the number of studies having focused on the dispositional procrastination–behavioral onset delay relation is very low, suggesting that this is a research area in need of increased focus. As argued, we believe that focus on onset delay is of particular interest. Although timeliness and lateness correlate highly with implemental delay (Svartdal and Steel, 2017), they are possibly more susceptible to cultural and contextual differences (Svartdal et al., 2016). This highlights the need for an analysis of behavioral procrastination that is less influenced by cultural and contextual differences. Focus on the timeliness of a finished task also often overlooks the fact that many forms of behavior are temporally distributed, one part being preparatory and often necessary for subsequent parts (e.g., Sheeran and Webb, 2016). Increased focus on delay early in such sequences may be more informative of the early phases of procrastination than focusing on the end product of the completed (or not completed) chain. A related issue concerns the way behavior has been conceptualized in procrastination studies. By addressing domain-specific behaviors described in a high-level language (e.g., Christmas shopping), time-related, low-level attributes of behavior may be easily overlooked. Low-level attributes of behavior (Vallacher and Wegner, 1987) address *how* actions are performed (e.g., in terms of tempo, speed, and onset delay) rather than *which* actions are performed. Because low-level attributes relate to the way actions are performed, and such attributes are rather difficult to describe and modulate verbally (Svartdal, 1995), they may provide information about procrastination that is not captured in current self-reported procrastination scales.

Finally, as seen in **Table 2**, several of the studies included self-reported dilatory behavior rather than objective measures of behavioral delay. Lane et al. (2013) examined a construct closely related to procrastination, impulsivity, and found that psychometric instruments focusing on this construct tended to correlate reliably with each other, whereas behavioral measures obtained through laboratory tests focusing on the same construct demonstrated lower within-tests correlations. Also, the psychometric scales correlated poorly with behavioral tests, even though they targeted the same construct. Such findings indicate that psychometric instruments may give an exaggerated impression of orderliness that does not match behavioral proxies well. For procrastination, this is particularly important, as behavioral delay is a defining characteristic of procrastination.

### The Present Studies

In the present studies, we examine various forms of behavioral delay related to the mechanisms discussed, focusing on implemental delay. Specifically, we address three facets of how behavioral delay expresses itself. Study 1 focused on delayed (as opposed to immediate) onset of intended behavior; Studies 2a and 2b addressed preferences for later rather than earlier, and Studies 3a and 3b addressed delay in preparatory behavior. Rather than looking at the overall delay in action implementation, we focus on delay in intentional behavior when an action possibility presents itself, either observed or as expressed in preferences.

We examine such delays in simplified and ecologically valid situations, that is, situations in which the observation of naturally occurring behavioral delay is possible. Thus, when a person is confronted with a simple choice situation with freely available alternatives, a preference for delay or actual behavioral delay displayed in that situation may be potentially informative of an underlying disposition to procrastinate. The overall model for the present thinking assumes that dilatory behavior as described above is a direct or indirect consequence of a “later” dictum and that such delay subsequently may be a contributing factor to the negative consequences seen in procrastination. Preferences and behavior were correlated with scores on procrastination instruments. The overall hypothesis is that people scoring relatively high on instrument measuring disposition procrastination adheres to a “later” dictum, and hence demonstrates behaviors and preferences accordingly.

## Study 1

As a direct exploration of the “later” dictum in implementing intended behavior, Study 1 observed people entering a shopping mall by escalators. When using an escalator, one has a simple choice: Remain still and let the escalator bring you up, or add speed by walking. In the present context, we assume that people opting for the first alternative adhere to a “wait” or “delay” rule, whereas people who select to walk do not. Hence, we expected that people standing still would demonstrate higher scores on a test for procrastination compared to walkers.

### Method

#### Participants

Participants were 56 adults (mean age = 38.5 years), 28 females. All were visitors to a shopping mall in northern Norway.

#### Procedure and Material

Observers (student assistants) were located at the upper end of different escalators in a shopping mall, being able to observe whether a potential participant walked versus did not walk while being on the escalator. People walking most or all of the escalator were classified as Walking; people standing still although being free to move (e.g., not hindered by a person standing in front; not carrying a heavy package) were classified as Standing. When leaving the escalator, each participant was approached by the assistant and asked to answer the Irrational Procrastination Scale (Steel, 2010). Participants filled in the questionnaire as well as age and gender information (paper and pencil). After completing these steps, the participant was thanked and given a short explanation of the study. Finally, the assistant coded the sheet as “S(tanding)” or “W(alking)” as well as gender and time of day (morning, noontime, evening). All information was given voluntarily and anonymously, and all participants gave informed consent to contribute after receiving brief information of the study.

#### Ethics

The current studies were part of a larger project that received ethical approval from the Regional Ethical Board in Tromsø, Norway (REK nord 2014/2313). Participation was voluntary, anonymous, and confidential. Participants were read a consent form describing the nature and purpose of the study and then provided informed consent before responding. No payment was provided.

### Results

An ANOVA with S(tand) versus W(alk) and gender as predictors and IPS score as the dependent measure revealed that walkers demonstrated lower IPS scores compared to people standing still (*M* = 2.45 vs. 2.88), *F*(1,50) = 4.926, *p* = 0.031, $\eta_{p}^{2}$ = 0.090. Also, the ANOVA indicated a significant interaction effect, *F*(1,50) = 7.586, *p* = 0.008, reflecting that walking women had lower IPS scores compared to men and also that standing women had higher IPS scores. The main effect of gender was not significant.

We also performed a separate ANOVA to test the potential effect of time (morning, noon, afternoon) on escalator behavior (Díaz-Morales et al., 2008). Here the ANOVA indicated a significant interaction effect of time, *F*(2,50) = 4.381, *p* = 0.018, reflecting that the stand versus walk effect primarily was visible in the afternoon, less so in the morning and at noon.

### Discussion

As predicted, people using an escalator to enter a shopping mall selected to walk or stand still in accord with their propensity to procrastinate. All participants shared the same overall intention to enter, yet differences appeared in the promptness of action implementation in accord with dispositional procrastination score. Although this difference may be influenced by a number of factors involved in goal striving (Steel et al., 2018), we believe that the important result in this context is that some people—procrastinators—chose to stand still. This response is consistent with a preference for delay when swift action is possible. In the present sample, this tendency was stronger in females compared to men and stronger in the afternoon compared to morning and noontime. Nonetheless, these results indicate that a simple choice behavior—stand versus walk—is related to procrastination score. As discussed (see **Table 1**), this automatic inclination to delay is consistent with an overall prediction that procrastinators delay action implementation.

## Study 2a

Study 2 explored the hypothesis that procrastination score as measured by a self-report instrument is related to time preferences in choice. If procrastinators adhere to a simple “later” dictum, behavior as well as choices involving time options (e.g., early vs. late) should be predictably related to procrastination score. This hypothesis was investigated in two different settings, training studio time preferences (i.e., preference for visiting the training studio early or late in the day) and seminar time preferences (i.e., preference for participating in seminars starting early vs. late in the day). The “later” dictum indicates that such preferences should be visible in that people visiting training studios late in the day should demonstrate higher procrastination scores (Study 2a) and that a similar difference should be observed in students participating in seminars later in the day (Study 2b).

### Method

#### Participants

Participants were 119 adults (mean age = 30.83 years), 59 females. All were visitors at three different training studios in a city in Northern Norway.

#### Procedure and Material

A student assistant approached visitors outside training centers early (9–12) or later in the day (12–15) and asked participants to answer the Irrational Procrastination Scale (IPS; Steel, 2010) as well as provide age and gender information (paper and pencil). Then, the assistant coded the sheet as “Early” or “Late.” All information was given voluntarily and anonymously, and all participants gave informed consent after receiving brief information about the study.

### Results

An overall ANOVA with IPS as dependent variable and time (early vs. late), gender, and three different training locations as predictors indicated a significant effect of time (*M*_Late_ = 2.79; *M*_Early_ = 2.46), *F*(1,105) = 6.668, *p* = 0.011, $\eta_{p}^{2}$ = 0.060. None of the other factors were significant, but one of the training locations demonstrated a lower overall IPS score in men compared to the two other locations, resulting in a significant location ^∗^ gender interaction, *F*(2,105) = 3.302, *p* = 0.040. This effect was not considered relevant for the present study.

### Discussion

As hypothesized, visitors at training centers early (09–12) versus late (12–15) demonstrated a significant difference in procrastination scores in the predicted direction. Although this difference is hypothesized to be a result of a dictum of “later” in people procrastinating more, this rule may have worked in direct ways as well as indirectly. Thus, on a given day, a procrastinating person may have selected training time later in the day although an earlier time slot was available (direct effect of a “later” rule). Alternatively, selection of later training time may be a consequence of time-related choices the day before, as in bedtime procrastination (Kroese et al., 2014), necessitating later training times the day after. Third, training time may have been occasioned by previously determined self-chosen time slots, reflecting a choice at an earlier occasion. Finally, training time may have been determined outside the person’s control and thus not informative of the person’s time preferences at all. Despite the noise introduced by the final possibility, the present data indicate that the behavior of choosing training times are predictably related to procrastination score, indicating that one or more of the procrastination-informative mechanisms are in operation.

These results are consistent with findings that procrastinators demonstrate a preference for eveningness (Díaz-Morales et al., 2008; Digdon and Howell, 2008; Hairston and Shpitalni, 2016). In the Díaz-Morales et al. (2008) study, participants completed the Early/Late Preference Scale (Smith et al., 2002) as well as two procrastination scales, the Adult Inventory of Procrastination (AIP; McCown et al., 1989) and the Decisional Procrastination Scale (DPS; Mann, 1982, unpublished). Díaz-Morales et al. (2008) reported a low but significant correlation between the AIP and the morningness-eveningness scale, *r* = -0.28, and a lower and non-significant correlation between the DPS and the morningness-eveningness scale. The present study extends these results by demonstrating that such time preferences are reflected in actual behavior.

## Study 2b

Although training at training centers is popular, only part of the population exercises, potentially restricting the external validity of the finding of Study 2a. Therefore, we conducted a second study with students enrolled in a large introductory course. As part of this course, seminar teaching was offered. At the beginning of the semester, students chose between available seminars, available at specific time slots throughout the day, with start times from 08.15 to 16.15. We expected that students selecting seminar times before versus after noon would do so at least in part as a reflection of the dictum “delay.” Hence procrastination scores should be higher in students selecting post-noon seminar times.

### Method

#### Participants

Participants were students (*N* = 140, 110 females) at an introductory course in psychology, recruited as part of an examination of procrastination instruments for an intervention study on procrastination (Nordby et al., 2016).

#### Procedure and Instruments

Questionnaires were distributed to registered students at seminars. All answered a procrastination scale, the Pure Procrastination Scale (PPS; Steel, 2010; Svartdal et al., 2016). This scale correlates highly, *r* = 0.87, with the IPS (Steel, 2010; Svartdal, 2015). As part of the questionnaire students reported which seminar group they attended, allowing for a grouping of seminars as “Early” (pre-noon) and “Late” (post-noon). We also asked participants to answer an additional question regarding typical bedtime on a weekday (21–00, 00–02, 02–05).

### Results

First, the ANOVA with PPS as the dependent variable and Early/Late and gender as predictors indicated a significant main effect of Early/Late, *M*_Early_ = 2.53 versus *M*_Late_ = 2.97, *F*(1,135) = 8.888, *p* = 0.003, $\eta_{p}^{2}$ = 0.062. The main effect of gender and the Early/Late ^∗^ gender interaction were not significant. Second, the mean PPS scores for participants indicating when they typically go to sleep were significantly different over typical bedtimes, *M*_21-00_ = 2.56, *M*_00-02_ = 2.98, *M*_02-05_ = 3.41, *F*(1,133) = 6.515, *p* = 0.002, $\eta_{p}^{2}$ = 0.089. In both cases, the main effect of gender was non-significant, as was the interaction effects.

### Discussion

These results demonstrate that preferred time for seminar attendance was predictably related to dispositional procrastination. As attendance time was determined at the start of the semester, procrastinators opting for seminars later in the day in accord with a “later” dictum. This result is again consistent with prior research on a preference for eveningness (Díaz-Morales et al., 2008) and may at least indirectly reflect reported bedtime preferences, which were also predictably related to procrastination level (cf. Kroese et al., 2014). Importantly, the “later” preference demonstrated here had specific behavioral consequences throughout the semester, illustrating how a simple and spontaneous decision at one occasion generates long-time consequences. The findings reported here are also consistent with results reported by Solomon and Rothblum (1984), who found that procrastinating students participating in experimental sessions tended to prefer sessions late in the semester. Similarly, Cassidy and Kangas (2014) showed that students signing up for a study on discounting behavior (measuring self-control/impulsivity) demonstrated a negative relation between self-control and time selected over the semester. Cassidy and Kangas (2014, p. 3) noted that “signing up earlier in the semester can be conceptualized as self-controlled activity,” and that signing up for late timeslots may be seen as a form of procrastination. The present data indicated a similar relation, albeit in time preferences over the day rather than the semester. Note that these results, as well as the Solomon and Rothblum (1984) and the Cassidy and Kangas (2014) results, go beyond an explanation in terms of morningness–eveningness, suggesting a simpler explanation in terms of a “later” rule with a possible origin in the mechanisms discussed in **Table 1**.

## Study 3a

Studies 1 and 2 demonstrated that procrastinators seem to adhere to the rule “later” when confronted with simple choice situations, resulting in a preference for options later rather than earlier. As the preferences demonstrated in these studies do not represent any specific disadvantages, this strategy may seem inconsequential. Thus, even if procrastinating students prefer late seminars, the outcome of attending late seminars may be just as high as attending seminars earlier in the day. Hence, for the “later” dictum to represent a disadvantage for procrastinators, detrimental consequences must be demonstrated. Here, bedtime procrastination (Kroese et al., 2014, 2016) may be a good model. First, a defining characteristic of bedtime procrastination is that the person fails to go to bed at the intended time, “while no external circumstances prevent a person from doing so.” Second, going to bed later than planned has potential negative short- and long-time consequences that demonstrate the disadvantage of such a habit.

In the same vein, we propose that other simple choices reflecting a preference for “later” may put the procrastinating person in a more disadvantageous position compared to non-procrastinators. Studies 3a and 3b focus on a possibly important example, how the “later” preference represents a financial disadvantage to the procrastinator. Here we address one of the delay mechanisms already discussed, delay in preparatory behavior. As preparatory behavior (e.g., preparing a shopping list) is distant from the actual planned behavior (shopping), we hypothesize that such preparatory behavior is easily delayed or even skipped by procrastinators. Hence, Study 3a examined lunch habits among students and employees in Norway. In this country, bringing your lunch is a long-standing tradition (ISIC, n.d.). Financially, this practice makes sense, as buying lunch at the cantina is much more expensive compared to the cost of bringing your lunch. However, as bringing your lunch requires planning and preparation of food before one leaves home, it is reasonable to assume that procrastinators delay this step, leaving home without food. Not planning lunch before leaving home may put the procrastinator in a disadvantageous situation at lunchtime. Clearly, if this habit continues over time, it will be financially disadvantageous to procrastinators.

### Method

#### Participants

Participants were students and employees at a Norwegian University (*N* = 123) at three different cantinas.

#### Procedure and Instruments

Student assistants approached visitors at the cantinas and asked them to fill in a shortened version of IPS (six items consistent with procrastination, a version found to be psychometrically equivalent to the full version; Svartdal and Steel, 2017). Upon completion of this task, the assistant coded whether the visitor had bought lunch in the cantina or brought his/her own. All participants agreed to participate, and all information was provided anonymously.

### Results

Initial analyses indicated that males reported higher procrastination levels compared to females, but the gender factor did not interact with the bring versus buy factor. An ANOVA with IPS score as the dependent variable and bring versus buy as the predictor indicated, as predicted, a significant difference between the groups, *M*_Bring_ = 2.92 versus *M*_Bought_ = 3.32, *F*(1,115) = 11.254, *p* = 0.001.

### Discussion

The present results demonstrated a difference in procrastination scores between cantina visitors bringing their food for lunch versus visitors buying their lunch, procrastinators ending up with the more expensive alternative. As the more expensive alternative was necessitated by not bringing a lunch package in the first place, a key to understanding the observed difference is related to an explanation of why respondents did not bring lunch from home. We propose a simple explanation in terms of impulsivity: Procrastinators are impulsive, and as planning and life organization correlate negatively with procrastination (e.g., Steel, 2007), it is understandable that procrastinators do not worry so much about events hours away. Thus, the present results may be seen as a consequence of procrastinators adhering to a “later” dictum, postponing the issue of lunch until lunchtime appears. In general, such a delay may be relatively inconsequential, but in Norway, such behavior will be quite costly, especially if it establishes itself as a habit.

## Study 3b

To substantiate the findings of Study 3a, we administered a survey focusing on personal finance, including a question on lunch habits.

### Method

#### Participants

Participants were 527 adults (377 females), mean age = 30.96 years (*SD* = 11.67). The majority of participants were employees (*n* = 253) and students (*n* = 194); the remaining 46 categorized themselves as “other.” All were recruited through social media (e.g., Facebook).

#### Materials and Procedure

Participants were invited to answer a web-based questionnaire^1^ containing questions about habits related to personal finance, procrastination (IPS; Steel, 2010), as well as age and gender. The questions about finance included (a) yearly income (five categories), (b) expectation for own economic situation in 1 year (five categories, 1 = *very bad* – 5 = *very good*), and (c) lunch habits (“usually bring my own lunch” and “usually buy in the cantina”). The questionnaire also contained other questions not included in the present study.

### Results and Discussion

A majority of the sample (*n* = 307) reported that they usually bring a lunch from home, the rest (*n* = 178) indicated that they usually buy in the cantina. An ANOVA with bring versus buy and gender as predictors indicated a significant lower mean IPS score in the Bring group, *M*_Bring_ = 2.71 (*SD* = 0.06) compared to the Buy group, *M*_Buy_ = 2.99 (*SD* = 0.07), *F*(1,446) = 10.271, *p* = 0.001, $\eta_{p}^{2}$ = 0.023. The effect of gender and gender ^∗^ bring/buy interaction were not significant. Thus, this result repeats the finding of Study 3a, albeit in self-report form.

Although buying food in the cantina is an expensive habit, it becomes increasingly detrimental for individuals with lower income levels. We therefore performed an ANOVA with bring versus buy and income level as predictors. The ANOVA indicated a significant main effect of bring (*M* = 2.65) versus buy (*M* = 3.00), *F*(1,442) = 20.901, *p* = 0.000, as well as a significant main effect of income levels, *F*(4,442) = 6.806, *p* = 0.000, reflecting an overall reduction in IPS scores with increasing income levels. The interaction effect was not significant, *F*(4,442) = 1.611, *p* = 0.170. The main effect of bring versus buy revealed itself primarily at lower income levels, indicating that people that would benefit the most from changing their expensive habit (procrastinators) suffer the most. A parallel analysis with financial situation expected in 1 year rendered very similar results.

These results demonstrate the importance of preparatory behavior in procrastination. This detrimental delay strategy may be encountered in many areas. For example, buying airline tickets long before traveling is often much cheaper than buying them a short time before traveling, putting the procrastinating airline passenger in a financially disadvantageous situation compared to non-procrastinating passengers. In the health domain, postponing vaccination may have negative effects (Baker, 2011). Finally, in the student domain, preparatory behaviors may be of potentially high significance, as the value of a given insight may be dramatically increased if it is acquired early rather than later. For example, understanding the concept of correlation early in a statistics course versus understanding it days before an exam implies very different learning benefits, even though outcome results as measured in examinations may not differ very much.

## General Discussion

The present paper hypothesized that procrastinators adhere to a simple behavioral rule—“later”—in common daily situations, resulting in predictable delays in behavioral onset, time preferences for “later” rather than “sooner,” and delayed preparatory behaviors with detrimental consequences later on. Such a “later” rule is connected to specific mechanisms assumed to mediate the relation between impulsiveness and behavioral delay. Examples of behavioral delays were given in observed or indirect form in five separate studies over a variety of situations. Common to these studies was that procrastination manifested itself behaviorally, predictably related to dispositional procrastination score. Overall, the present results, summarized in **Table 3**, provide examples of behavioral delays related to dispositional procrastination, confirming the overall assumption that higher procrastination is associated with behavioral onset delay. These studies provided this evidence in situations rarely previously studied, thus extending the procrastination phenomenon to new domains. More importantly, the present studies focused on time-related, low-level attributes of behavior rather than domain-specific, high-level behaviors. As such low-level behavioral attributes are rather difficult to modulate by rules (Svartdal, 1995), actual behavior may in such cases be more informative about procrastination than self-reported habits and behaviors. Also, as such low-level behaviors are difficult to describe by the actor, they may cause delays in ways that occur largely unnoticed and hence are difficult to report, for example in procrastination self-report measures. Increased focus on low-level aspects of behavior may, therefore, be important both in the understanding of procrastination and in self-reported procrastination.

**Table 3:** Results, present studies.

	Variables	*M* (95% confidence intervals)	
Study 1	Standing vs. walking in escalator; IPS	*M*_Stand_	2.89 (2.59–3.18)
		*M*_Walk_	2.41 (2.14–2.67)
Study 2a	Early vs. late visitors to training studio; IPS	*M*_Early_	2.48 (2.30–2.66)
		*M*_Late_	2.79 (2.68–3.01)
Study 2b	Early vs. late seminar preference; PPS	*M*_Early_	2.46 (2.30–2.63)
		*M*_Late_	2.87 (2.70–3-04)
Study 3a	Bringing vs. buying lunch; IPS (6 items)	*M*_Bring_	2.92 (2.77–3.07)
		*M*_Buy_	3.32 (3.14–3.51)
Study 3b	Self-reported bring vs. buy lunch; IPS	*M*_Bring_	2.67 (2.58–2.76)
		*M*_Buy_	2.98 (2.86–3.10)

*Results are for main effects of predictor variables. IPS, Irrational Procrastination Scale; PPS, Pure Procrastination Scale.*

The present studies addressed delay in the implementation of planned behavior. Such delay, often named the intention-action delay, constitute a core attribute of procrastination (Steel, 2010). Most studies that have examined behavioral delay have focused on lateness/timeliness in completing the intended behavior (e.g., Lay, 1986; Tice and Baumeister, 1997; Howell et al., 2006). In contrast, the present studies focused on action onset delay and preferences for onset delay. Most planned action requires sustained performance over time, completing it being dependent on a number of factors that may or may not be under control of the actor. By focusing on delay in intended behavior as expressed when action possibility presents itself, either as observed action or implemental intentions expressed in preferences, the present studies address on an important and under-investigated part of the procrastination problem. The fact that the behavioral delay examples included in the present studies all were freely chosen, minimally influenced by contextual or cultural factors, as well as non-reflective, indicate that procrastinators are inclined to delay when an action possibility presents itself.

Focus on the initial delay of planned action, rather than on timeliness/lateness, may be important in understanding the procrastination problem and the problems unnecessary delay brings on the procrastinator. Such delays may manifest themselves as lingering or hesitation once an action possibility occurs (Study 1), a preference for later rather than sooner (Studies 2a and 2b), and delay in behavior necessary for subsequent behavior (Studies 3a and 3b), as well as in other ways. Such implemental delay may contribute to negative consequences over time. For example, delaying the onset of planned behavior decreases the time-window for completion, which may negatively affect performance and increase stress (Tice and Baumeister, 1997). Early onset of implemental action may also have beneficial psychological consequences, even if implementation is not completed. For example, having started the implementation of some planned project (e.g., reading a book, writing an essay, painting the house) turns an abstract intention into something concrete, thereby facilitating execution of planned action (e.g., McCrea et al., 2008). Even if the task is not finished, having started it may increase rather than decrease motivation to re-engage (Reeve et al., 1986). Finally, getting an early start on some project may change motivation, with self-perceptions shifting from “not doing = not interested” to “doing = interested” (Bem, 1972). In sum, instigating rapid implementation of intentions may prevent many of the negative behavior inclinations observed in procrastination. Techniques that help people in formulating and realizing their intentions (e.g., Gollwitzer and Sheeran, 2006; Sheeran and Webb, 2016), may, therefore, be of prime importance in reducing and preventing procrastination.

## Future Studies

A common theme to the studies of this paper, as well as in the definition of procrastination and its measurement, is that the procrastinator delays when prompt action is possible and preferable. This contrasts with the conception of procrastination as an impulsive person (van Eerde, 2003; Steel, 2007), preferring immediate rather than delayed outcomes. Why, then, do procrastinators seem to follow a “delay” rule when the opposite is possible and preferable? We have discussed a number of mechanisms that may mediate the impulsivity–delay relation (cf. **Table 1**), and the overall effect of these mechanisms seems to be a simple “delay” rule. Future research should explore this relation more thoroughly to determine additional mechanisms. The long-time effect of escaping and avoiding aversive events are of particular interest. Escaping or avoiding aversive tasks simply by stopping action (take a break) or avoiding the situation may relieve stress and induce a better mood in the short run (Baumeister et al., 1994; Tice and Bratslavsky, 2000). However, the immediacy of rewarding consequences from such strategies points to a potentially very powerful mechanism in generating and sustaining procrastination because diversion or passivity is effectively reinforced. Hence, a long history of escaping or avoiding aversive situations by simply doing nothing (i.e., passivity) may give passivity secondary reinforcement properties in just the same way as effort associated with reward can acquire secondary reinforcement properties (Eisenberger, 1992). Accordingly, delay, hesitation, and lingering may be activities that are reinforcing to the procrastinator, and hence may be hypothesized to represent a hedonically attractive outcome that is always available for the procrastinating person. Such a mechanism may help explain the relation between procrastination and the passivity seen in depressed individuals as well as in everyday procrastination. If true, passivity is a continuously available reward for the procrastinator, and increasingly so as the procrastination habit is getting more firmly established. Clearly, if delayed onset is a characteristic of procrastination, increased focus on various forms of such delay is of interest, both in terms of understanding procrastination and in prevention and intervention measures.

In the studies reported in this paper, dilatory behavior was predictably related to dispositional measures of procrastination. This is reassuring and demonstrates that dispositional measures are informative of behavioral inclinations, albeit in relatively crude form. However, as self-reported procrastination lacks a calibration mechanism that may differentiate between trivial but harshly judged procrastination and more serious forms (e.g., Gröpel and Steel, 2008; Rozental and Carlbring, 2014; Svartdal and Steel, 2017), more work is needed in developing measures, both behavioral and in self-report form, that may assist in such calibration efforts. Here, objective behavior-focused measures of procrastination may represent important supplements to self-report measures.

## Author Contributions

FS collected the data, ran analyses, and wrote the draft. SG and FF participated in discussions and in editing the document.

## Conflict of Interest Statement

The authors declare that the research was conducted in the absence of any commercial or financial relationships that could be construed as a potential conflict of interest.

## Footnotes

1 www.qualtrics.com

## References

[B1] Akerlof G. A. (1991). Procrastination and obedience. Washington, DC.

[B2] Baker L. (2011). Vaccinate. Don’t procrastinate. 15 6–9.

[B3] Baumeister R. F. Heatherton T. F. Tice D. M. (1994). *Losing Control: How and Why People Fail at Self-Regulation*. San Diego, CA: Academic Press.

[B4] Bem D. J. (1972). Self-perception theory. 6 1–62. 10.1016/S0065-2601(08)60024-6

[B5] Byrne A. Dowd K. Blake D. P. Cairns A. J. G. (2006). There’s no time like the present: the cost of delaying retirement saving. 15 213–231.

[B6] Cassidy R. N. Kangas B. D. (2014). Impulsive students participate later: delay discounting in a research subject pool. 30 1–5.

[B7] Chen B. B. Chang L. (2016). Procrastination as a fast life history strategy. 14 1–5. 10.1177/1474704916630314

[B8] Clariana M. Gotzens C. Badia D. M. Cladellas R. (2012). Procrastination and cheating from secondary school to university. 10 737–754. 27899829

[B9] Díaz-Morales J. F. Ferrari J. R. Cohen J. R. (2008). Indecision and avoidant procrastination: the role of morningness—eveningness and time perspective in chronic delay lifestyles. 135 228–240. 10.3200/GENP.135.3.228-240 18649490

[B10] Digdon N. L. Howell A. J. (2008). College students who have an eveningness preference report lower self-control and greater procrastination. 25 1029–1046. 10.1080/07420520802553671 19005903

[B11] Eisenberger R. (1992). Learned Industriousness. 99 248–267. 10.1037/0033-295X.99.2.248 1594725

[B12] Gollwitzer P. M. Sheeran P. (2006). Implementation intentions and goal achievement: a meta-analysis of effects and processes. 38 69–119. 10.1016/S0065-2601(06)38002-1 18096108

[B13] Gröpel P. Steel P. (2008). A mega-trial investigation of goal setting, interest enhancement, and energy on procrastination. 45 406–411. 10.1016/j.paid.2008.05.015

[B14] Gustavson D. E. Miyake A. Hewitt J. K. Friedman N. P. (2014). Genetic relations among procrastination, impulsivity, and goal-management ability: implications for the evolutionary origin of procrastination. 25 1178–1188. 10.1177/0956797614526260 24705635 PMC4185275

[B15] Hairston I. S. Shpitalni R. (2016). Procrastination is linked with insomnia symptoms: the moderating role of morningness-eveningness. 101 50–56. 10.1016/j.paid.2016.05.031

[B16] Howell A. J. Watson D. C. Powell R. A. Buro K. (2006). Academic procrastination: the pattern and correlates of behavioural postponement. 40 1519–1530. 10.1016/j.paid.2005.11.023

[B17] ISIC (n.d.). Available at: https://www.isic.no/studentliv/7-ting-som-kjennetegner-norske-studenter-i-utlandet/

[B18] Klingsieck K. B. (2013). Procrastination: when good things don’t come to those who wait. 18 24–34. 10.1027/1016-9040/a000138

[B19] Krause K. Freund A. M. (2014). Delay or procrastination–A comparison of self-report and behavioral measures of procrastination and their impact on affective well-being. 63 75–80. 10.1016/j.paid.2014.01.050

[B20] Kroese F. M. De Ridder D. T. Evers C. Adriaanse M. A. (2014). Bedtime procrastination: introducing a new area of procrastination. 5:611. 10.3389/fpsyg.2014.00611 24994989 PMC4062817

[B21] Kroese F. M. Evers C. Adriaanse M. A. de Ridder D. T. (2016). Bedtime procrastination: a self-regulation perspective on sleep insufficiency in the general population. 21 853–862. 10.1177/1359105314540014 24997168

[B22] Lane S. D. Cherek D. R. Rhoades H. M. Pietras C. J. Tcheremissine O. V. (2013). Relationships among laboratory and psychometric measures of impulsivity: implications in substance abuse and dependence. 2 33–40. 10.1097/00132576-200302020-00001

[B23] Lay C. H. (1986). At last, my research article on procrastination. 20 474–495. 10.1016/0092-6566(86)90127-3

[B24] Mann L. Burnett P. Radford M. Ford S. (1997). The Melbourne decision making questionnaire: an instrument for measuring patterns for coping with decisional conflict. 10 1–19. 10.1002/(SICI)1099-0771(199703)10:1<1::AID-BDM242>3.0.CO;2-X

[B25] McCown W. Johnson J. Petzel T. (1989). Procrastination, a principal components analysis. 10 197–202. 10.1016/0191-8869(89)90204-3

[B26] McCrea S. M. Liberman N. Trope Y. Sherman S. J. (2008). Construal level and procrastination. 19 1308–1314. 10.1111/j.1467-9280.2008.02240.x 19121142

[B27] Moon S. M. Illingworth A. J. (2005). Exploring the dynamic nature of procrastination: a latent growth curve analysis of academic procrastination. 38 297–309. 10.1016/j.paid.2004.04.009

[B28] Nordby K. Wang C. E. A. Dahl T. I. Svartdal F. (2016). Intervention to reduce procrastination in first-year students: preliminary results from a Norwegian study. 3:e10 10.15714/scandpsychol.3.e10

[B29] Patrzek J. Sattler S. van Veen F. Grunschel C. Fries S. (2015). Investigating the effect of academic procrastination on the frequency and variety of academic misconduct: a panel study. 40 1014–1029. 10.1080/03075079.2013.854765

[B30] Reeve J. Cole S. G. Olson B. C. (1986). The Zeigarnik effect and intrinsic motivation: Are they the same? 10 233–245. 10.1007/BF00992318

[B31] Roig M. DeTommaso L. (1995). Are college cheating and plagiarism related to academic procrastination? 77 691–698. 10.2466/pr0.1995.77.2.691

[B32] Rozental A. Carlbring P. (2014). Understanding and treating procrastination: a review of a common self-regulatory Failure. 5 1488–1502. 10.4236/psych.2014.513160

[B33] Schouwenburg H. C. (1995). “Academic procrastination: Theoretical notions, measurement, and research,” in eds Ferrari J. R. Johnson J. L. (New York, NY: Plenum Press) 71–96. 10.1007/978-1-4899-0227-6_4

[B34] Senécal C. Lavoie K. Koestner R. (1997). Trait and situational factors in procrastination: an interactional model. 12 889–903.

[B35] Sharma L. Markon K. E. Clark L. A. (2014). Toward a theory of distinct types of “impulsive” behaviors: a meta-analysis of self-report and behavioral measures. 140 374–408. 10.1037/a0034418 24099400

[B36] Sheeran P. Webb T. L. (2016). The intention–behavior gap. 10 503–518. 10.1111/spc3.12265

[B37] Sirois F. M. (2007). “I’ll look after my health, later”: a replication and extension of the procrastination–health model with community-dwelling adults. 43 15–26. 10.1016/j.paid.2006.11.003

[B38] Sirois F. M. (2014). Procrastination and stress: exploring the role of self-compassion. 13 128–145. 10.1080/15298868.2013.763404

[B39] Smith C. S. Folkard S. Schmieder R. A. Parra L. F. Spelten E. Almiral H. (2002). Investigation of morning–evening orientation in six countries using the preferences scale. 32 949–968. 10.1016/S0191-8869(01)00098-8

[B40] Solomon L. J. Rothblum E. D. (1984). Academic procrastination: frequency and cognitive–behavioral correlates. 31 503–509. 10.1037/0022-0167.31.4.503 14567164

[B41] Stead R. Shanahan M. J. Neufeld R. W. (2010). “I’ll go to therapy, eventually”: procrastination, stress and mental health. 49 175–180. 10.1016/j.paid.2010.03.028

[B42] Steel P. (2007). The nature of procrastination: a meta-analytic and theoretical review of quintessential self-regulatory failure. 133 65–94. 10.1037/0033-2909.133.1.65 17201571

[B43] Steel P. (2010). Arousal, avoidant and decisional procrastinators: Do they exist? 48 926–934. 10.1016/j.paid.2010.02.025

[B44] Steel P. Brothen T. Wambach C. (2001). Procrastination and personality, performance, and mood. 30 95–106. 10.1016/S0191-8869(00)00013-1 22442254

[B45] Steel P. Ferrari J. (2013). Sex, education and procrastination: an epidemiological study of procrastinators’ characteristics from a global sample. 27 51–58. 10.1002/per.1851

[B46] Steel P. Svartdal F. Thundiyil T. Brothen T. (2018). Examining procrastination across multiple goal stages: a longitudinal study of temporal motivation theory. 9:327. 10.3389/fpsyg.2018.00327 29666590 PMC5891720

[B47] Steel P. Weinhardt J. (2017). “The building blocks of motivation,” in 2nd Edn Vol. 3 eds Anderson N. Ones D. S. Sinangil H. K. Viswesveran C. (Thousand Oaks, CA: Sage).

[B48] Svartdal F. (1995). When feedback contingencies and rules compete: testing a boundary condition for verbal control of instrumental performance. 26 221–238.

[B49] Svartdal F. (2015). Measuring procrastination: psychometric properties of the Norwegian versions of the irrational procrastination scale (IPS) and the pure procrastination scale (PPS). 61:18 10.1080/00313831.2015.1066439

[B50] Svartdal F. Pfuhl G. Nordby K. Foschi G. Klingsieck K. B. Rozental A. (2016). On the measurement of procrastination: comparing two scales in six European countries. 7:1307. 10.3389/fpsyg.2016.01307 27630595 PMC5005418

[B51] Svartdal F. Steel P. (2017). Irrational delay revisited: examining five procrastination scales in a global sample. 8:1927. 10.3389/fpsyg.2017.01927 29163302 PMC5676095

[B52] Tice D. M. Baumeister R. F. (1997). Longitudinal study of procrastination, performance, stress, and health: the costs and benefits of dawdling. 8 454–458. 10.1111/j.1467-9280.1997.tb00460.x

[B53] Tice D. M. Bratslavsky E. (2000). Giving in to feel good: the place of emotion regulation in the context of general self-control. 11 149–159. 10.1207/S15327965PLI1103_03

[B54] Tice D. M. Bratslavsky E. Baumeister R. F. (2001). Emotional distress regulation takes precedence over impulse control: If you feel bad, do it! 80 53–67. 10.1037/0022-3514.80.1.53 11195891

[B55] Vallacher R. R. Wegner D. M. (1987). What do people think they’re doing? Action identification and human behavior. 94 3–15. 10.1037/0033-295X.94.1.3

[B56] van Eerde W. (2003). A meta-analytically derived nomological network of procrastination. 35 1401–1418. 10.1016/S0191-8869(02)00358-6

[B57] van Hooft E. A. Born M. P. Taris T. W. Van der Flier H. Blonk R. W. (2005). Bridging the gap between intentions and behavior: implementation intentions, action control, and procrastination. 66 238–256. 10.1016/j.jvb.2004.10.003

[B58] Worthley D. L. Cole S. R. Esterman A. Mehaffey S. Roosa N. M. Smith A. Turnbull D. Young G. P. (2006). Screening for colorectal cancer by faecal occult blood test: why people choose to refuse. 36 607–610. 10.1111/j.1445-5994.2006.01155.x 16911554

